# Pretreatment with Gemcitabine/5-Fluorouracil Enhances the Cytotoxicity of Trastuzumab to HER2-Negative Human Gallbladder Cancer Cells In Vitro and In Vivo

**DOI:** 10.1155/2019/9205851

**Published:** 2019-03-25

**Authors:** Wei Wang, Zhenhua Hu, Yu Huang, Huilin Zheng, Qiang Sun, Qifan Yang, Yuan Zhang, Linshi Zhang, Weilin Wang

**Affiliations:** ^1^Department of Hepatobiliary and Pancreatic Surgery, The Second Affiliated Hospital, School of Medicine, Zhejiang University, China; ^2^Key Laboratory of Precision Diagnosis and Treatment for Hepatobiliary and Pancreatic Tumor of Zhejiang Province, The First Affiliated Hospital, School of Medicine, Zhejiang University, China; ^3^Clinical Research Center of Hepatobiliary and Pancreatic Diseases of Zhejiang Province, The First Affiliated Hospital, School of Medicine, Zhejiang University, China; ^4^Department of Gastrointestinal Surgery, Shaoxing People's Hospital (Shaoxing Hospital, Zhejiang University School of Medicine), Shaoxing 312000, Zhejiang, China

## Abstract

The effects of standard clinical therapies including surgery and chemotherapy are poor in advanced gallbladder cancer (GBC). There are a few reported cases of human epidermal growth factor receptor 2 (HER2)-positive GBC that responded well to trastuzumab. But trastuzumab has not yet been used to treat HER2-negative GBC. In this study, we investigated the cytotoxic effects of different combined therapies with trastuzumab and gemcitabine and/or 5-fluorouracil on HER2-negative GBC cell lines in vitro and in vivo. Trastuzumab alone showed almost no cytotoxicity to GBC cells with originally low HER2 gene amplification. Sequential therapy with chemotherapy followed by trastuzumab showed superiority over reverse sequential chemotherapy (*P*<0.05), concurrent combined chemotherapy (*P*<0.05), chemotherapy alone (*P*<0.05), and trastuzumab alone (*P*<0.05) in terms of cytotoxicity. Sequential therapy with chemotherapy followed by trastuzumab nearly completely inhibited cell viability in HER2-negative GBC cells. Similar results were observed with regard to apoptosis. Western blot analysis showed that gemcitabine/5-fluorouracil increased the expressions of total and phosphorylated forms of HER2, thus enhancing the cytotoxicity of trastuzumab. In vivo study verified the results of in vitro study by terminal deoxynucleotidyl transferase-mediated deoxyuridine triphosphate nick-end labeling assay and immunohistochemical analysis. Moreover, not only the lightest tumor bearing but also the best survival state was detected in sequential therapy with chemotherapy followed by trastuzumab group compared with other groups. Our in vivo and in vitro data suggest that sequential therapy with gemcitabine/5-fluorouracil followed by trastuzumab represents a novel and promising therapeutic strategy against HER2-negative GBC. The upregulation of phosphorylated HER2 and phosphorylated-AKT induced by gemcitabine/5-fluorouracil treatment shows that HER2/AKT pathway is triggered.

## 1. Introduction

Gallbladder cancer (GBC), the most common biliary tract malignancy and the sixth most common digestive tract cancer worldwide [1], is a highly fatal disease with poor prognosis [2]. It has an incidence of 0.0025% [3]. 12,360 new cases and 3,960 deaths of GBC are estimated in the United States in 2019 by the American Cancer Society [4]. Owing to its unspecific symptoms and signs, early diagnosis of GBC is difficult. The overall mean survival period for GBC patients is only 6 months [5]. Up to now, the standard clinical treatments for GBC are surgery and chemotherapy. However, neither is satisfactory. Novel therapeutic modalities are urgently needed to improve clinical efficacy, especially for patients with unresectable GBC.

Human epidermal growth factor receptor 2 (HER2) is a transmembrane glycoprotein with tyrosine kinase receptor activity. It provides signal transduction for cell proliferation, adhesion, migration, apoptosis, and differentiation [6–8] and plays a role in the development of breast [9] and gastric cancers [10]. HER2-targeted therapy has already been proven to improve the prognosis of patients with breast and gastric cancers [10, 11]. Kiguchi et al. confirmed that overexpression of HER2 protein in gallbladder epithelium leads to the development of cancer in transgenic mice [12]. However, its biological importance or clinical implications in GBC are unclear.

Trastuzumab, a monoclonal antibody directed to the extracellular HER2 receptor, has been approved in the treatment of advanced HER2-positive breast cancers [13]. It is also recommended for use in advanced HER2-positive gastric cancers [10, 14]. The application of trastuzumab in combination with chemotherapeutic drugs in the treatment of patients with HER2-positive breast or gastric cancer is more effective than standard chemotherapy [10, 15]. Although there are a few reported cases of HER2-positive GBC that responded well to trastuzumab [16], combined therapy with chemotherapy and trastuzumab has not yet been identified as a standard therapy for GBC. The optimal combination and order of cytotoxic drugs needed to achieve greatest efficacy are still unclear. Furthermore, relevant in vivo and in vitro research are very limited. Finally, HER2-targeted therapy is only recommended for HER2-positive rather than HER2-negative patients.

The present study was undertaken to investigate whether combined therapy with chemotherapy and trastuzumab in HER2-negative GBC cell lines in vitro and in vivo could improve cytotoxicity, and which combination would achieve the best efficacy. In addition, differences in apoptosis, cell cycle, and HER2/protein kinase B (AKT) signaling pathway were analyzed to demonstrate the reasons behind different effects.

## 2. Materials and Methods

### 2.1. Cell Lines, Reagents, and Antibodies

NOZ cell line was a gift from Prof. Yingbin Liu at Xinhua Hospital affiliated to Shanghai Jiaotong University School of Medicine. GB-D1 cell line was purchased from CELLBIO, Inc. (Shanghai, China). Gemcitabine (GEM, G) was purchased from JARI Pharmaceutical Co. (Lianyungang, China). 5-Fluorouracil (5-Fu, F) was purchased from Nanjing Oddo Foni Biology Technology Co. (Nanjing, China). Trastuzumab (Herceptin, H) was purchased from Genentech, Inc. (South San Francisco, USA). Primary antibodies against HER2, phosphorylated-HER2 (Tyr1248), and phosphorylated-AKT (Ser473) were purchased from Cell Signaling Technology, Inc. (Beverly, USA). The anti-AKT antibody and anti-phospho-HER2 (Y1248) antibody were purchased from R&D Systems, Inc. (Minnesota, USA).

The human GBC cell lines NOZ and GB-D1 were cultured in Dulbecco's modified Eagle medium (DMEM) with high glucose containing 10% fetal bovine serum, streptomycin (100 *µ*g/mL), and penicillin (100 U/mL). Cells were incubated in an atmosphere with 5% CO2 at 37°C.

### 2.2. Detection of HER2 Gene Amplification in GBC Cells Using Dual-Color Fluorescence In Situ Hybridization (FISH)

GBC cells were detached using trypsin-ethylenediaminetetraacetic acid (EDTA), washed with phosphate buffered saline (PBS), and dehydrated with methanol/acetic acid. Cells were placed on slides and baked at 56°C for 3 h. The slides were placed in 2× saline sodium citrate (SSC) for 5 min and then dehydrated in 70, 80, and 100% ethanol for 3 min each. After natural drying, 10 *µ*L hybridization mixture was used with the SpectrumGreen probe for centromere 17 (CEP17) and the SpectrumOrange probe specific for the HER2 gene. The slides were then incubated for 5 min at 75°C, followed by 14-18 h at 38°C. The slides were then rinsed in a 2× SSC/0.1% Nonidet P-40 solution (Sigma-Aldrich, Bornem, Belgium) for 3 min at room temperature and then in 0.4× SSC/0.3% Nonidet P-40 solution for 3 min at 75°C. Once again, the slides were placed in 2× SSC for 5 min and then dehydrated in 70, 80, and 100% ethanol for 3 min each. After natural drying, 10 *µ*L 4,6-diamidino-2 phenylindole dihydrochloride (DAPI) was used to counterstain nuclei. HER2 signals and CEP17 signals were scored in 100 nuclei.

### 2.3. Cell Viability Assay

A Cell Counting Kit-8 (Dojindo, Japan) was used to measure cell viability, and optical density (OD) value was read at 450 nm. Approximately GBC 2.5×10^4^ cells/mL were seeded in 96-well plates overnight. The experimental group was divided into five subgroups according to different treatments, including trastuzumab only (H), chemotherapy only (G/F), sequential therapy with chemotherapy followed by trastuzumab (G/F→H), concomitant therapy with chemotherapy and trastuzumab (H+G/F), and sequential therapy with trastuzumab followed by chemotherapy (H→G/F). For the sequential therapy with chemotherapy followed by trastuzumab group, GBC cells were incubated in DMEM containing GEM (NOZ, 345 *µ*g/mL and GB-D1, 2258 *µ*g/mL) and/or 5-Fu (NOZ, 0.024 *µ*g/mL and GB-D1, 123 *µ*g/mL) for 48 h first, then washed with PBS, and finally treated with trastuzumab (NOZ, 40 *µ*g/mL, and GB-D1, 0.44 *µ*g/mL) for 48 h. For the sequential therapy with trastuzumab followed by chemotherapy group, GBC cells that were pretreated with trastuzumab were further treated with chemotherapeutic drugs. For the concomitant therapy of chemotherapy and trastuzumab group, GBC cells were treated with chemotherapeutic drugs and trastuzumab simultaneously. The concentration of each drug in single therapy was the same as that in combination therapy, and the experiment was repeated three times.

### 2.4. Apoptosis and Cell Cycle Analysis

GBC cells were seeded at a density of 2.5×10^4^ cells/mL in 6-well plates overnight and treated with various drugs. They were then detached using trypsin-EDTA and rinsed twice with PBS. To investigate apoptosis, cells were stained with a fluorescein isothiocyanate (FITC)-conjugated annexin V apoptosis detection kit (Dojindo, Japan) according to the manufacturer's protocol and then analyzed via flow cytometry (BD, USA). For cell cycle analysis, cells were fixed with absolute alcohol at 4°C overnight. The fixed cells were centrifuged to remove ethanol, washed with PBS, and stained with DNA prep stain (Beckman Coulter, USA) at 4°C for 30 min in the dark. Finally, data were also analyzed via flow cytometry (BD, USA).

### 2.5. Western Blot Analysis

After treatment under desired conditions, GBC cells were harvested and cellular proteins were extracted. Protein concentrations were determined using a Pierce BCA Protein Assay Kit (Thermo Scientific, USA) according to the manufacturer's protocol. Equal amounts of cellular proteins were separated by electrophoresis in 10% gels (Beyotime Institute of Biotechnology, Shanghai, China) and transferred onto polyvinylidene fluoride (PVDF) membranes (Schleicher & Schuell GmbH, USA). After blocking with 5% blocking buffer for 60 min, membranes were immunostained with primary antibodies against HER2, phosphorylated-HER2 (Tyr1248), AKT, phosphorylated-AKT (Ser473), and *β*-actin (Sigma-Aldrich) at 4°C overnight followed by incubation with secondary antibodies at 20°C for 60 min. Protein bands were visualized in an electrochemiluminescence (ECL) image system.

### 2.6. Establishment of the Xenograft Mouse Model and Drug Tests

Female BALB/c nude mice (SLACCAS, shanghai, China) were allowed to acclimatize for one week before the study. A suspension of NOZ cells (4×10^6^ cells/mouse) was injected subcutaneously in the right flank of each mouse. Ten days later, all mice bore a tumor of approximately 200–400 mm^3^ in volume. Tumor-bearing mice were randomly allocated into different treatment groups and a control group. Each group consisted of eight to nine mice (Table 1). There was no statistic difference of body weight between groups before the treatment. Mice were intraperitoneally (i.p.) injected of normal saline (20mg/kg) or GEM (100mg/kg) [17] or 5-Fu (30mg/kg) [18] and/or trastuzumab (20mg/kg) [19] according to the schedule of Table 1. Mice body weight and tumor volume were measured every three days. The tumor volume was calculated using the following formula: AB2/2 (A is the longer diameter. B is the shorter diameter.) [17, 19]. Mice were killed on day 18. Tumors were harvested for further experiments. All animal experiments were approved by the Animal Experimental Ethical Inspection of the First Affiliated Hospital, Zhejiang University School of Medicine (Agreement No. 2018-753).

### 2.7. Terminal Deoxynucleotidyl Transferase-Mediated Deoxyuridine Triphosphate Nick-End Labeling (TUNEL) Assay

Apoptotic response of tumor tissues embedded in paraffin wax was evaluated using a One Step TUNEL Apoptosis Assay Kit (Beyotime Institute of Biotechnology, Shanghai, China) according to the manufacturer's instructions. Nuclear DNA was counterstained with DAPI (Beyotime Institute of Biotechnology, Shanghai, China). All photographs were captured under a Nikon ECLIPSE TS100 microscope (Nikon Corp., Tokyo, Japan). Ten optical areas, containing 1,000 cells at least, were counted in each slide under a magnification of ×200 (objective ×20 and ocular ×10). The proportion of apoptotic cancer cells was calculated as apoptosis rate.

### 2.8. HER2 Immunohistochemical (IHC) Analysis

Tumor tissues were stained using the StreptAvidin-Biotin Complex DAB Assay Kit (Solarbio Corp., Beijing, China) according to the manufacturer's protocol and counterstained with hematoxylin. IHC score of HER2 was calculated as described previously [20] at a magnification of ×200.

### 2.9. Statistical Analysis

Statistical calculations were performed using SPSS 17.0 statistical software. Comparisons among multiple groups were performed by one-way analysis of variance (ANOVA). Statistical significance was defined as P < 0.05.

## 3. Results

### 3.1. FISH

**Figure 1:**
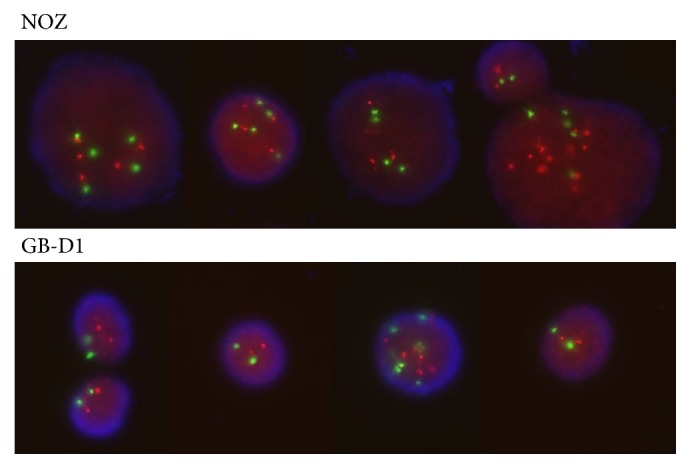
*Microphotographs of dual-color FISH for HER2 detection in GBC cell lines (×1,000). *SpectrumGreen probe for centromere 17 and SpectrumOrange probe for HER2 gene. The HER2:CEP17 ratio in NOZ and GB-D1 cell lines was 1.2 and 1.08, respectively.

The HER2:CEP17 ratio in NOZ and GB-D1 cell lines was 1.2 and 1.08, respectively (Figure 1). Currently, there are no HER2 diagnostic criteria for GBC, and we therefore referred to breast cancer criteria. Cancer cells are defined as HER2 gene amplification-positive when the ratio of average HER2 copy number to that of CEP17 exceeds 2. Therefore, both GB-D1 and NOZ cells were classified as HER2-negative cells [21].

### 3.2. Synergistic Effects of Trastuzumab and Chemotherapeutic Drugs

**Figure 2:**
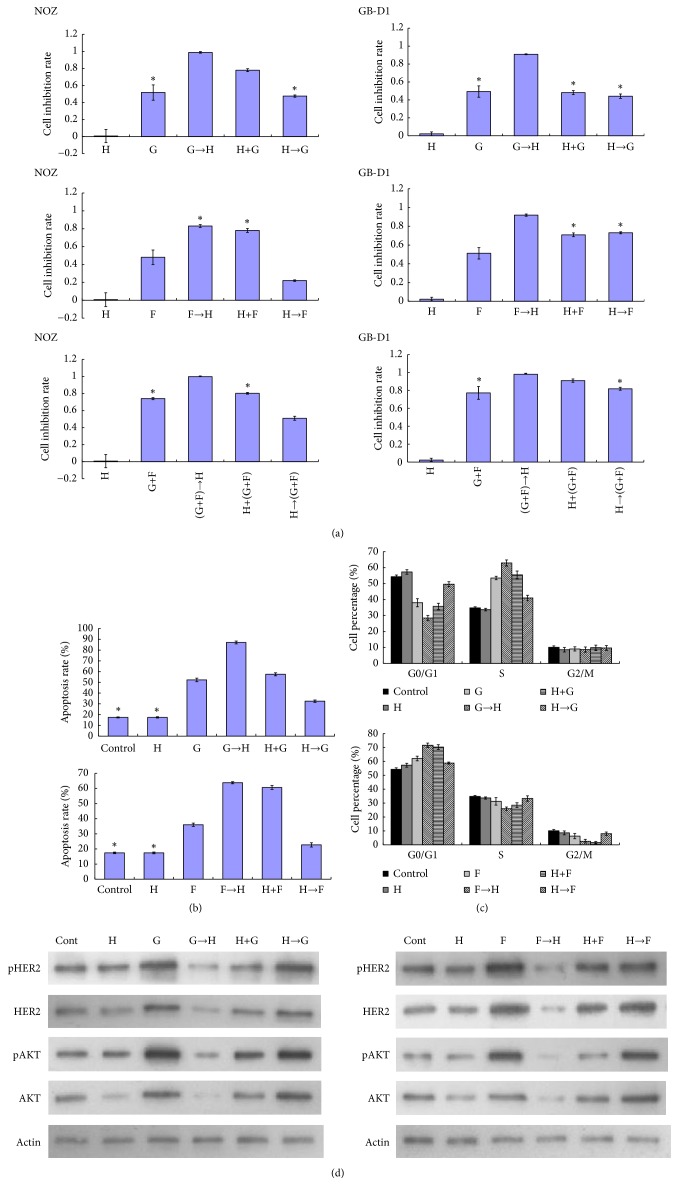
*Cell viability inhibition rates and apoptosis in GBC cell lines after treatment with various drugs*. (a) Cell viability inhibition rates in NOZ cells and GB-D1 cells after treatment with trastuzumab (Herceptin, H) only, chemotherapy (GEM, G and 5-Fu, F) only, sequential therapy with chemotherapy followed by trastuzumab, concomitant therapy with chemotherapy and trastuzumab, and sequential therapy with trastuzumab followed by chemotherapy were evaluated. Sequential therapy with chemotherapy followed by trastuzumab demonstrated superiority over the others. *∗*P > 0.05. Others: P < 0.05. Cell viability inhibition rate was calculated as follows: (nontreated cells OD - treated cells OD) / (nontreated cells OD - blank OD). (b) Results relating to apoptosis in NOZ cells were similar to those of the cell viability assay. Sequential therapy with chemotherapy followed by trastuzumab demonstrated superiority over the others in terms of apoptosis. *∗*P > 0.05. Others: P < 0.05. (c) Effects of trastuzumab and/or GEM on cell cycle in NOZ cells: P > 0.05, G versus (H + G) in the G1 phase of the cell cycle; control versus H, and G versus (H + G) in the S phase of the cell cycle, all in the G2 phase of the cell cycle. Others: P < 0.05. Effects of trastuzumab and/or 5-Fu on cell cycle in NOZ cells: P > 0.05: H versus (H→F), and (H + F) versus (F→H) in the G1 phase of the cell cycle; control versus H, control, H or F versus (H→F) in the S phase of the cell cycle; control versus H, H versus F, (F→H) versus (H + F), control, H or F versus (H→F) in the G2 phase of the cell cycle. Others: P < 0.05. (d) Western blots were performed to detect the effects of trastuzumab and/or chemotherapeutic drugs on the expressions of key proteins HER2, pHER2, AKT, and pAKT in the HER2/AKT signaling pathway in NOZ cells. *β*-actin was used as a loading control. NOZ cells showed increases in pHER2, pAKT, HER2, and AKT expression following G/F alone or H→G/F treatment.

Cell lines were treated with trastuzumab and chemotherapeutic agents including GEM and/or 5-Fu in different orders. As shown in Figure 2(a), trastuzumab showed synergistic cytotoxicity when concurrently combined with GEM or 5-Fu alone in NOZ cells, whereas synergistic cytotoxicity was observed when concurrently combined with 5-Fu alone or GEM plus 5-Fu in GB-D1 cells. In addition, sequential therapy with chemotherapy followed by trastuzumab nearly completely inhibited cell viability in both NOZ and GB-D1 cells. However, sequential therapy with trastuzumab followed by chemotherapy showed unstable cytotoxicity when collocated with different chemotherapeutic drugs and is unsuitable for use in HER2-negative GBC cells. Moreover, GEM and/or 5-Fu alone seem to be more potent in terms of cytotoxicity than trastuzumab. Trastuzumab alone showed almost no cytotoxic effects on HER2-negative GBC cells, but pretreatment with GEM and/or 5-Fu enhanced the cytotoxicity of trastuzumab. The experiment was repeated three times with similar results.

### 3.3. Cell Apoptosis and Cell Cycle Arrest

The results of cell apoptosis investigation were similar to those of the cell viability assay. GEM and 5-Fu both promoted apoptosis of NOZ cells. The combination of trastuzumab and chemotherapeutic drugs increased apoptosis compared to chemotherapy alone. When treated with chemotherapy followed by trastuzumab, cell apoptosis was further increased (Figure 2(b)). In addition, trastuzumab blocked cells in G1 phase and 5-Fu increased the G1 population. However, S arrest was more predominant in NOZ cells treated with GEM (Figure 2(c)).

### 3.4. GEM/5-Fu Upregulates HER2 Expression and Enhances the Cytotoxicity of Trastuzumab In Vitro

GEM or 5-Fu alone increased the expression of total and phosphorylated forms of HER2 and AKT, the key molecules of the HER2/AKT signaling pathway in NOZ cells with originally low HER2 gene amplification. Thus, the cytotoxic effects of trastuzumab were enhanced by downregulating increased HER2/AKT pathway signaling in GEM/5-Fu-pretreated NOZ cells (Figure 2(d)). This mechanism may contribute to the synergistic cytotoxic effects of sequential therapy with chemotherapy followed by trastuzumab on GBC.

### 3.5. GEM/5-Fu Enhances the Cytotoxicity of Trastuzumab In Vivo

**Figure 3:**
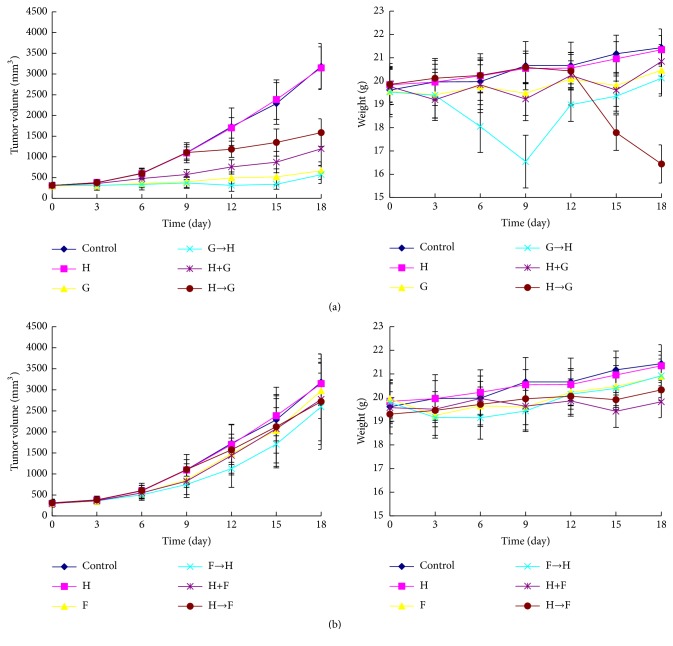
*Line charts of mice body weight and tumor volume after treatment with various drugs*. Treatment was started ten days after tumor inoculation. Mice were intraperitoneally injected of normal saline (20mg/kg) or GEM (100mg/kg) or 5-Fu (30mg/kg) and/or trastuzumab (20mg/kg) according to the schedule of Table 1. Mice body weight and tumor volume were measured every three days. On day 18, tumors were harvested. (a) On day 18, the tumor volume of G→H group was the smallest (P < 0.05: G→H group versus other groups expect for G group). Moreover, G→H group's body weight was heavier than that of H→G group (P < 0.05), but not statistically different compared with G group and H+G group (P > 0.05). In addition, H group had similar tumor volume and body weight compared with the control group (P > 0.05). (b) A similar tendency was also observed in F→H group compared with other groups, but not so obviously as G→H group (P > 0.05).

**Figure 4:**
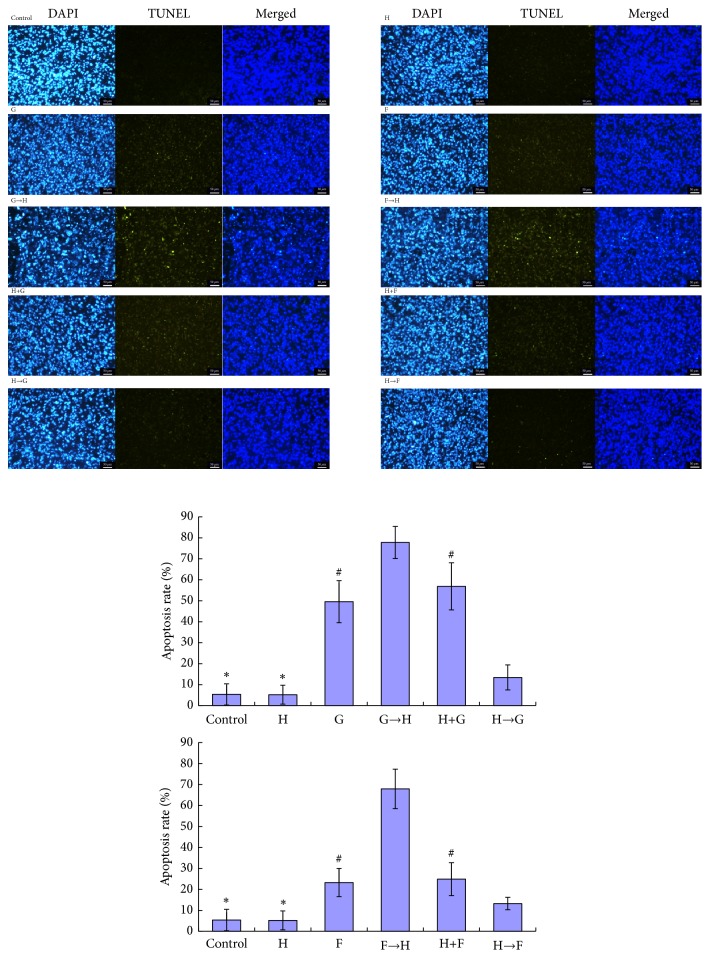
*Cell apoptosis of tumor tissues in the xenograft mouse model by TUNEL assay (magnification of ×200)*. There were statistic differences between groups, except for the groups with the same mark, such as *∗* or #. Apoptosis rate of G→H group or F→H group was higher than those of other groups (P < 0.05).

**Figure 5:**
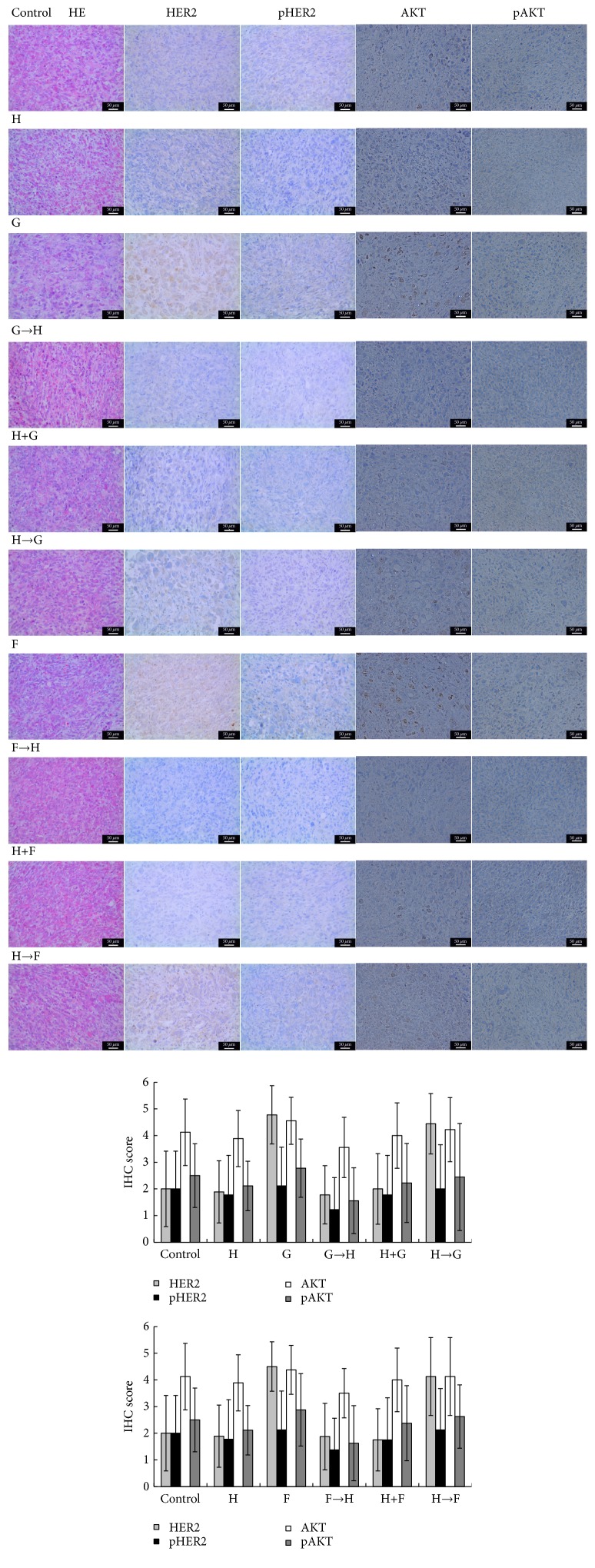
*Key protein expressions of HER2/AKT signaling pathway in tumor tissues of the xenograft mouse model by IHC assay (magnification of ×200)*. GEM or 5-Fu alone increased the expression of HER2 protein. HER2 IHC scores of group G, H→G, F, and H→F were higher than those of other groups (P < 0.05). But there were no statistic differences between other groups.

The synergistic antitumor activities of trastuzumab and chemotherapeutic drugs were detected in the NOZ xenograft model. In this study, trastuzumab alone group had similar tumor volume and body weight compared with the control group. The main reason for weight gain of control group and trastuzumab group in the later phase of the study was the amplification of tumor. At the end of the study, we found that the general state of G→H group was the best in all groups, including the spirit and appetite. Moreover, subcutaneous fat of G→H group was also the thickest. Although the body weight of G→H group was not the heaviest in all groups, it was heavier than that of H→G group significantly (P < 0.05) and was not statistically different compared with G group and H+G group (P > 0.05) (Figure 3). Most of all, the tumor volume of G→H group was the smallest (P < 0.05, except for G→H verus G). On day 18, A similar tendency was also observed in F→H group compared with other groups, but not so obviously as G→H group (P > 0.05). Moreover, apoptosis rate of G→H group or F→H group was higher than those of other groups by TUNEL assays (P < 0.05) (Figure 4). In other words, sequential therapy with chemotherapy followed by trastuzumab markedly increased cell apoptosis compared with chemotherapy alone and other combined chemotherapies. In addition, IHC analysis demonstrated that GEM or 5-Fu alone increased the expression of HER2 protein (Figure 5). IHC scores of group G, H→G, F, and H→F were higher than those of other groups (P < 0.05).

## 4. Discussion

Conventional chemotherapeutic drugs for GBC include GEM and 5-Fu. GEM is a nucleoside analog that has long been used as the basis of GBC treatment. Additionally, 5-Fu is also an inhibitor of DNA synthesis. Both GEM and 5-Fu can inhibit cell proliferation and promote apoptosis. Although GEM and 5-Fu are partially effective, their applications are limited by their cumulative dose-dependent toxicities, which include myelotoxicity, nephrotoxicity, vomiting, and nausea. Trastuzumab-based combination chemotherapy is a novel therapeutic strategy with better efficacy, lower drug dosage, and fewer side effects.

Conventional chemotherapy for GBC is less than ideal. To improve the prognosis of GBC, the establishment of new, promising treatment is essential. Nam et al. [17] have proved that trastuzumab can be combined with GEM to treat HER2-positive GBC cells effectively. The cytotoxicity of trastuzumab in HER2-amplified GBC cell lines is similar to that in HER2-positive breast or gastric cancer cells. However, the study was just limited to HER2-positive cells, and the experimental group did not involve sequential therapy with chemotherapy and trastuzumab. Our preclinical data indicate that sequential therapy with chemotherapy followed by trastuzumab is superior to both concomitant therapy and sequential therapy with trastuzumab followed by chemotherapy in terms of cytotoxicity. Remarkably, trastuzumab alone showed almost no cytotoxicity in NOZ and GB-D1 cells with originally low HER2 gene amplification. However, the cytotoxicity of trastuzumab was effectively enhanced in sequential therapy with chemotherapy followed by treatment with trastuzumab.

Results from the analysis of apoptosis were similar to those of the cell viability assay. The combination of trastuzumab and chemotherapeutic drugs induced apoptosis to a greater extent than chemotherapy alone, and apoptosis was further increased in cells treated with chemotherapy followed by trastuzumab. These results indicate that the drug's effect lies primarily in inducing apoptosis. In addition, trastuzumab tended to increase G1 arrest. 5-Fu blocked cells in G1 phase, while GEM increased the S population in NOZ cells.

To further evaluate the potential therapeutic effect of sequential therapy with chemotherapy followed by trastuzumab, NOZ xenograft model was established for drug tests in vivo. In this in vivo study, trastuzumab alone showed no cytotoxicity to GBC compared with normal saline. The main reason for weight gain of control group and H group in the later phase was the amplification of tumor. And thin subcutaneous fat proved that mice were emaciated and malnourished. At the end of the study, we found that the general state of G→H group was the best in all groups, including the spirit and appetite. Moreover, subcutaneous fat of G→H group was also the thickest. Although the body weight of G→H group was not the heaviest in all groups, it was heavier than that of H→G group significantly (P < 0.05) and was not statistically different compared with G group and H+G group (P > 0.05). Most of all, the tumor volume of G→H group was the smallest (P < 0.05, except for G→H versus G), meaning that the main factor of weight gain in G→H group was not the tumor growth. Generally speaking, not only the lightest tumor bearing but also the best survival state was detected in G→H group compared with other groups. F→H was similar to G→H, but not so obviously (P > 0.05). TUNEL assay showed that apoptosis rate of G→H group or F→H group was higher than those of other groups (P < 0.05). In other words, sequential therapy with chemotherapy followed by trastuzumab showed superiority over chemotherapy alone and other combined chemotherapies in terms of cell apoptosis, suggesting that sequential therapy with chemotherapy followed by trastuzumab may be a novel and promising therapeutic strategy against HER2-negative GBC.

In this study, GEM or 5-Fu alone increased the expressions of HER2 and AKT in NOZ cells with originally low HER2 gene amplification. There may be four reasons to explain this phenomenon. Firstly, both GEM and 5-Fu play anticancer roles mainly by inhibiting DNA synthesis, suggesting that upregulation of HER2 and AKT may be associated with DNA synthesis inhibition. Secondly, GEM/5-Fu may promote transcription and/or translation of HER2/AKT by some mechanisms. For instance, microRNAs were reported to regulate HER2 translation [22]. But it is not clear whether GEM/5-Fu regulates HER2 translation by microRNAs. Thirdly, Kan S et al. reported that BAY11-7082, a nuclear factor-kappaB inhibitor, suppressed HER2 upregulation following GEM treatment in breast cancer [23]. Nuclear factor-kappaB may play a role in HER2 upregulation induced by GEM. Finally, GEM/5-Fu may inhibit HER2 degradation [24].

pHER2, the activated form of HER2 [25], may indicate activated HER2 signaling. HER2 phosphorylation results in intracellular signaling and activation of several genes involved in cell growth [26]. In breast cancer, the expression of Tyr1248-pHER2 was significantly associated with total HER2 expression and was a more specific marker for HER2 gene amplification than total HER2 expression [21]. HER2- or pHER2-positive cancers, especially Tyr1248-pHER2-positive cancers, tend to be in a more advanced stage [21] and are associated with poorer prognosis [25, 27] than pHER2-negative tumors. In the present study, when used alone, both GEM and 5-Fu increased expression of the total and phosphorylated forms of HER2 and AKT, the key molecules of the HER2/AKT signaling pathway in NOZ cells with originally low HER2 gene amplification, thus enhancing the cytotoxic effects of trastuzumab by downregulating the enhanced HER2/AKT pathway of GEM/5-Fu-pretreated NOZ cells. In other words, sequential therapy with chemotherapy followed by trastuzumab increased HER2/AKT expressions firstly and then increased cleavage in the HER2/AKT signaling pathway. This mechanism may contribute to the synergistic cytotoxic effects of sequential therapy with chemotherapy followed by trastuzumab on GBC.

HER2 amplification or overexpression is observed in 15–20% of breast cancers [28, 29], 12.5–17% of gastric cancers [30, 31], and 16.6% of GBCs [32] approximately. HER2-targeted therapy is only recommended for HER2-positive patients. Up to now, the use of trastuzumab had not yet been suggested in HER2-negative cases. Therefore, according to the current recommended uses of trastuzumab, most GBC patients cannot benefit from trastuzumab because of the low HER2 expression. If HER2 expression can be promoted ahead, more HER2-negative patients with GBC will benefit from HER2-targeted therapy. Previous reports on induced expression of HER2 are rather limited. Shin Kan. et al. reported that GEM enhances HER2 expression in pancreatic ductal adenocarcinoma cells [24] and breast cancer cells [23]. However, preclinical evidence of chemotherapy-mediated upregulation of HER2 expression in HER2-negative GBC cells has not yet been reported. Our in vitro and in vivo data indicate that HER2 upregulation by GEM and/or 5-Fu enhances the cytotoxic effects of trastuzumab. This study provided preclinical evidence for the application of sequential therapy with chemotherapy followed by trastuzumab in HER2-negative GBC for the first time.

In conclusion, this study innovatively used trastuzumab to treat HER2-negative GBC cells. Trastuzumab alone showed almost no cytotoxic effects on GBC cells with originally low HER2 gene amplification. GEM or 5-Fu upregulated HER2 expression in HER2-negative GBC cells, thus enhancing the cytotoxic effects of trastuzumab by downregulating the enhanced HER2/AKT pathway in GEM/5-Fu-pretreated GBC cells. Among the various combined therapies tested, sequential therapy with chemotherapy followed by trastuzumab showed superiority over chemotherapy alone and other combined chemotherapies in terms of cytotoxicity to HER2-negative GBC cells, suggesting that sequential therapy with chemotherapy followed by trastuzumab represents a novel and promising therapeutic strategy against HER2-negative GBC.

## Figures and Tables

**Table 1:** Schedule of treatment with various drugs in each group.

Group	Day 0	Day 3	Day 6	Day 9	Day 12	Day 15	Day 18
Control(n=8)	NS	-	NS	-	NS	-	-
H(n=9)	H	-	H	-	H	-	-
G(n=9)	G	-	G	-	G	-	-
G→H(n=9)	G	G	G	H	H	H	-
H+G(n=9)	H+G	-	H+G	-	H+G	-	-
H→G(n=9)	H	H	H	G	G	G	-
F(n=8)	F	-	F	-	F	-	-
F→H(n=8)	F	F	F	H	H	H	-
H+F(n=8)	H+F	-	H+F	-	H+F	-	-
H→F(n=8)	H	H	H	F	F	F	-

The control group was intraperitoneally injected of normal saline. The dosages of various drugs using in treatment groups were as follows: normal saline, NS (20mg/kg, i.p.); GEM, G (100mg/kg, i.p.); 5-Fu, F (30mg/kg, i.p.); and Herceptin, H (20mg/kg, i.p.).

## Data Availability

The data used to support the findings of this study are included within the article and the supplementary information file (available here). More details are available from the first author [Wei Wang, wwsjd@163.com].

## Abbreviations

GBC: Gallbladder cancer
HER2: Epidermal growth factor receptor 2
AKT: Protein kinase B
GEM, G: Gemcitabine
5-Fu, F: 5-Fluorouracil
Herceptin, H: Trastuzumab
DMEM: Dulbecco's modified Eagle medium
FISH: Fluorescence in situ hybridization
EDTA: Ethylenediaminetetraacetic acid
TUNEL: Transferase-mediated deoxyuridine triphosphate nick-end labeling
IHC: Immunohistochemical
ANOVA: Analysis of variance
PBS: Phosphate buffered saline
SSC: Saline sodium citrate
CEP17: Centromere 17
OD: Optical density
FITC: Fluorescein isothiocyanate
PVDF: Polyvinylidene fluoride
ECL: Electrochemiluminescence.

## Supplementary Materials

Supplementary Materials Detection of half maximal inhibitory concentrations (IC50) of herceptin (H), GEM (G), and 5-Fu (F) in Gallbladder cancer(GBC) cell lines including NOZ and GB-D1 cells using cell viability assay: GBC cells were incubated in Dulbecco's modified Eagle medium containing GEM, 5-Fu, or Herceptin with different concentrations for 24h and 48h, respectively. Cell inhibition rate was detected using cell viability assay. As shown in the figures, 24h is not long enough to inhibit cell viability in vitro. Herceptin alone showed almost no cytotoxic effects on NOZ and GB-D1 cells. Moreover, IC50 of GEM for 48 h is 345 *µ*g/mL in NOZ cells and 2258 *µ*g/mL in GB-D1 cells, respectively, while IC50 of 5-Fu for 48 h is 0.024 *µ*g/mL in NOZ cells and 123 *µ*g/mL in GB-D1 cells, respectively.
