# Supplementary material for: Pretreatment with Gemcitabine/5-Fluorouracil Enhances the Cytotoxicity of Trastuzumab to HER2-Negative Human Gallbladder Cancer Cells In Vitro and In Vivo
Source: Biomed Res Int. 2019 Mar 25;2019:9205851. doi: 10.1155/2019/9205851 (PMC6452559; doi:10.1155/2019/9205851)
Supplement: Supplementary Materials — Detection of half maximal inhibitory concentrations (IC50) of herceptin (H), GEM (G), and 5-Fu (F) in Gallbladder cancer(GBC) cell lines including NOZ and GB-D1 cells using cell viability assay: GBC cells were incubated in Dulbecco's modified Eagle medium containing GEM, 5-Fu, or Herceptin with different concentrations for 24h and 48h, respectively. Cell inhibition rate was detected using cell viability assay. As shown in the figures, 24h is not long enough to inhibit cell viability in vitro. Herceptin alone showed almost no cytotoxic effects on NOZ and GB-D1 cells. Moreover, IC50 of GEM for 48 h is 345 µg/mL in NOZ cells and 2258 µg/mL in GB-D1 cells, respectively, while IC50 of 5-Fu for 48 h is 0.024 µg/mL in NOZ cells and 123 µg/mL in GB-D1 cells, respectively. [file 9205851.f1.docx]

NOZ

GB-D1

**Detection of half maximal inhibitory concentrations (IC50) of herceptin (H), GEM (G) and 5-Fu (F) in GBC cell lines using cell viability assay.**

IC50 of GEM for 48 h is 345 µg/mL in NOZ cells and 2258 µg/mL in GB-D1 cells, respectively. While IC50 of 5-Fu for 48 h is 0.024 µg/mL in NOZ cells and 123 µg/mL in GB-D1 cells, respectively.
